# Laparoscopic adrenalectomy - is it safe in hands of residents in training?

**DOI:** 10.1186/s12894-019-0538-5

**Published:** 2019-10-28

**Authors:** Jadwiga Dworak, Michał Wysocki, Anna Rzepa, Michał Natkaniec, Michał Pędziwiatr, Andrzej Budzyński, Piotr Major

**Affiliations:** 2nd Department of General Surgery, Jagiellonian University Medical College, Kopernika 21 St., 31-501 Kraków, Poland

**Keywords:** Laparoscopic adrenalectomy, Adrenal gland, Resident, Learning curve, Operator conversion, Intraoperative difficulties, Complications

## Abstract

**Background:**

Laparoscopic adrenalectomy (LA) has become the “gold standard” for treating most adrenal tumors in the past decade. However, it is still considered a relatively complicated procedure requiring experience from surgeon. The aim of the study was to evaluate the safety of laparoscopic adrenalectomy performed by residents who are undergoing training in general surgery.

**Methods:**

A prospectively collected database containing all 300 transperitoneal laparoscopic adrenalectomies performed in II Department of General Surgery JU MC, Krakow between January 2013 and March 2018 was retrospectively reviewed. Patients were divided into two groups; patients operated on by residents (group 1, 54 operations) and by attending general surgeons (group 2, 246 operations). We compared the course of the operation and patient hospitalization in these two groups. If the operation was completed by a different person than the one who started the procedure, we refer to this as “operator conversion”.

**Results:**

We found no differences in demographic factors or comorbidities between the two groups. The mean operative time was similar in the residents’ and the specialists’ groups (*p* = 0.5761). Median blood loss did not differ between the groups (*p* = 0.4325). The overall ratio of intraoperative adverse events was similar in both groups (*p* = 0.8643). The difference in the ratio of perioperative complications between the groups was not statistically significant (*p* = 0.6442). The average mean hospital stay after surgery was 2 days for both groups. We identified 25 cases (8.33%) of operator conversion; the difference in operator conversions between two groups was not statistically significant (*p* = 0.1741).

**Conclusions:**

Laparoscopic transperitoneal adrenalectomy performed by a supervised resident is a safe procedure. The course of the operation and patient hospitalization did not differ importantly when comparing procedures performed by residents and attending surgeons. Liberal use of operator conversions from resident to attending surgeon and from a surgeon to a senior surgeon provides reasonable safety and prevents complications. In high-volume centers performing minimally invasive techniques, closed supervision allows residents to safely perform LA.

## Background

Laparoscopic adrenalectomy (LA) has become the “gold standard” for treating most adrenal tumors in recent years [1]. Nevertheless, LA is considered an advanced procedure with a potential risk of serious complications requiring a high skill level [2–5]. Surgical residents today face the challenge of learning complex skills in a limited time, growing expectations regarding efficiency, quality, and financial viability of health care delivery. Minimally invasive techniques became more popular and available and were therefore included in surgery residents’ training programs [6]. However, it remains unclear whether advanced procedures like LA can be performed as safely by residents as by attending surgeons. Previous studies have addressed the interaction between residents’ participation in surgical procedures and outcomes such as morbidity and mortality with conflicting results. Differences in the results likely relate to the variability of research questions, study settings, research strategies and outcome measures. Furthermore, most of these studies addressed this issue indirectly and failed to correctly adjust for potential confounders [7–9].

The aim of this study was therefore to assess the safety of LA performed by residents who are undergoing training in general surgery.

## Methods

We performed a retrospective analysis of prospectively collected data of patients who underwent surgical treatment of adrenal tumors in our academic teaching hospital and tertiary referral center between January 2013 and March 2018. The inclusion criterion was laparoscopic transperitoneal adrenalectomy for adrenal tumors. The indication for surgery was either a hormonally active tumor, or, in the case of non-secreting incidentaloma, size ≥40 mm, rapid growth in follow-up studies, or a so-called “*radiological malignant phenotype*” of the tumor. Exclusion criteria were patients undergoing open adrenalectomy, adrenalectomy as a part of multiorgan resection, bilateral adrenalectomy, adrenalectomy from a posterior approach.

All patients underwent preoperative imaging studies (ultrasound, computed tomography, magnetic resonance imaging, or, if necessary, positron emission computed tomography). Tumor size was estimated in imaging studies (computed tomography or magnetic resonance imaging). Prior to surgery, a routine panel of laboratory tests was conducted to establish the tumor’s hormonal activity. In cases of suspected pheochromocytoma, patients were preoperatively treated with alpha-blockers (doxazosin 20 mg/day and additional beta-blockers in case of co-existing tachycardia). The operative method of choice in our department is laparoscopic transperitoneal lateral total adrenalectomy, which is performed similarly to its description elsewhere [10, 11]. In this paper, we understood the acronym to mean laparoscopic transperitoneal adrenalectomy.

The team that performed the surgeries comprised six operators, including four attending surgeons (five surgeons with 2–5 years of specialization during their inclusion to the study and one senior surgeon who was experienced in laparoscopic procedures) and two surgery residents (in their fourth to sixth years of training). Patients were divided into two groups. The first group (Group 1) was operated on by a resident and the second group (Group 2) by a attending general surgeon. Prior to attempting first adrenalectomy, the residents in training were required to acquire an appropriate theoretical background in endocrine surgery and experience in laparoscopic surgery (including obligatory structured training on simulators at the Department of Medical Education, Jagiellonian University Medical College). Every resident had to assist in at least 15 LAs, after which they could perform procedures supervised and assisted by a surgeon with expertise in adrenal gland surgery.

The study analyzed the following characteristics: operative time, mean blood loss, number of intraoperative adverse events, intraoperative difficulties and operator conversions, conversion rate, perioperative complications, reoperations, readmissions, length of hospital stay and 30-day-mortality rate.

If the operation was completed by a different person than the one who started the procedure, we refer to this as “operator conversion”. The reasons for such conversions were lack of progress in the procedure, anatomical difficulties imposing a high risk of complications and intraoperative adverse events. When residents were operating, the supervising surgeon took over the operation. For patients operated on by attending surgeons, operator conversions to the senior surgeon with the highest expertise in laparoscopic adrenalectomy were included in the analysis. The number of and reasons for such operator conversions were assessed.

Directly after each procedure, every main operator was obligated to note down the intraoperative difficulties, which were defined as surgeon-reported obstacles during the operation. The surgeon had to set all reported obstacles into an LA Matrix Table Questionnaire. The table comprised the following groups: difficulty in achieving good and sufficient working space, intra-abdominal adhesions or anatomy obstructing surgery, difficulty in dissection, difficulty in recognizing the anatomy or the proper layers, difficulty in achieving sufficient hemostasis and the need for assistance from a supervisor (operator conversion).

Intraoperative adverse events were defined as any iatrogenic adverse events during the operation that were not derived from the standard LA technique. Perioperative complications were defined as adverse events occurring within 30 days after the procedure. Major complications were defined as grade III or higher in Clavien-Dindo classification [12]. Intraoperative blood loss was measured from the amount of blood aspirated in the suction machine. Excessive intraoperative blood loss was defined as a loss of more than 500 ml. The operative time was measured from the skin incision to its closure. The length of hospital stay (LOS) was defined as the time in days from surgery until discharge from hospital.

**Fig. 1:**
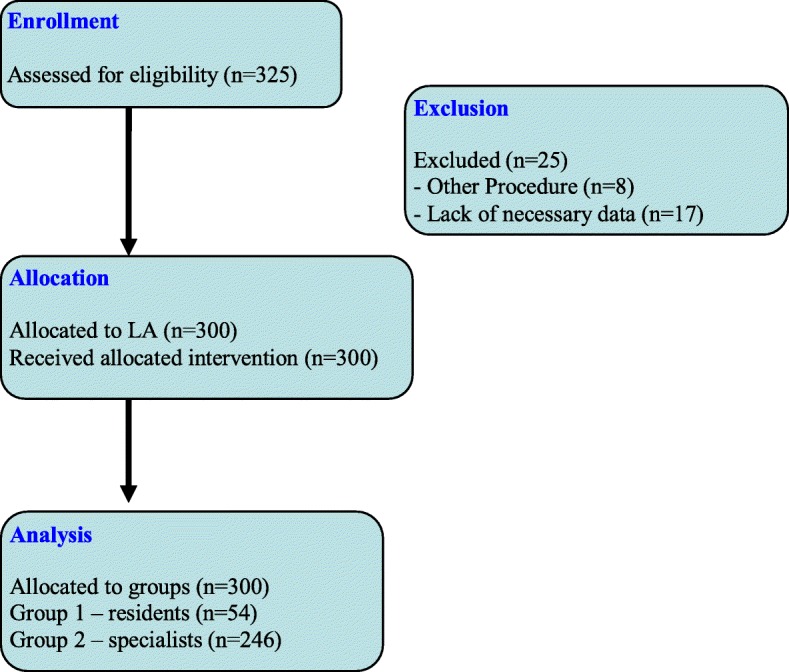
The flow of patients through the study

All 300 consecutive patient operated on between January 2013 and March 2018 were included in the study [181 (60.33%) females, 119 (39.67%) males, with a mean age of 57.7 years (19–87 years, SD ± 13.1 years)]. The flow of patients through the study is shown in Fig. 1.

There were 150 left-sided and 150 right-sided procedures. The indication for surgery was a hormonally inactive tumor in 144 (48.0%) patients, a catecholamine-secreting tumor in 58 (19.3%) patients, a glucocorticosteroid-secreting tumor in 30 (10.0%) patients, an aldosterone-producing tumor in 21 (7.0%) patients, a virilizing tumor in 3 (1.0%) patients and a cancer or a metastasis to the adrenal gland in 44 (14.7%) patients. The mean size of the removed lesion was 45 mm (6–150 mm, SD ± 21.7 mm).

We found no statistically significant differences in demographic factors or comorbidities between the two groups. Demographic data of the analyzed groups are shown in Table 1.

**Table 1:** Characteristics of studied groups

Parameter	All	Group 1 (surgical residents)	Group 2 (attending surgeons)	*p* value
Number of patients, n (%)	300	54	246	–
Females, n (%)	181 (60.33%)	29 (53.70%)	152 (61.79%)	0.2715
males, n (%)	119 (39.67%)	25 (46.30%)	94 (38.21%)	
Mean age (years, ± SD)	57.72 ± 13.10	58.74 ± 12.12	57.50 ± 13.32	0.5352
Mean BMI (kg/m2, ± SD)	28.19 ± 5.44	28.02 ± 4.65	28.23 ± 5.60	0.8062
Mean size (cm, ± SD)	4.50 ± 2.17	4.26 ± 1.96	4.55 ± 2.22	0.3881
Side of the procedure				
-right	150	28	122	0.7638
-left	150	26	124	
Type of the tumor				
-hormonally inactive	144	23	121	0.7238
-catecholamine-secreting	58	11	47	
-glucocorticosteroid-secreting	30	5	25	
-aldosterone-producing	21	6	15	
-virilizing	3	0	3	
-cancer or metastasis	44	9	35	

Statistical analysis was performed using StatSoft Statistica version 12.0 PL (StatSoft Inc., Tulsa, OK, USA). The results are presented as mean ± standard deviation (SD), median and interquartile range and odds ratios (ORs) with 95% confidence intervals when appropriate. Tests were used according to the type of variable. Groups were compared using the χ^2^ test for categorical variables. Because of a lack of normal data distribution, continuous variables were assessed using the Mann–Whitney U test. Results were considered statistically significant when the *p*-value was less than 0.05.

All procedures involving human participants were performed in accordance with the ethical standards of the institutional and national research committee and with the 1964 Helsinki Declaration and its later amendments or comparable ethical standards [13]. Approval by the local ethics review committee - Komisja Bioetyczna Uniwersytetu Jagiellońskiego was obtained (nr 1072.6120.88.2018).

## Results

Out of 300 operations included in this analysis 54 were performed by residents and 246 by attending surgeons. The mean operative time was 103.5 min (range 30–350, SD ± 43.5). The mean operative time of LA was similar in the residents’ and the specialists’ groups. Median blood loss was 50 ml and did not differ between the groups. Intraoperative adverse events were observed in 26 (8.67%) operations (Table 2). There were five events in Group 1 and 21 events in Group 2. We observed more than one intraoperative adverse event in three patients. The overall intraoperative adverse events ratios were similar in both groups.

**Table 2:** Intraoperative adverse events

Parameter	Group 1 (residents)	Group 2 (specialists)	*p* –value
Total	5 (9.26%)	21 (8.54%)	0.8643
Tumour capsule rupture	1 (1.85%)	1 (0.41%)	0.7960
Vessel injury	2 (3.70%)	5 (2.03%)	0.8112
Other organ injury	1 (1.85%)	8 (3.25%)	0.9158
Excessive intraoperative blood loss	1 (1.85%)	10 (4.07%)	0.7011

The incidence of the intraoperative difficulties, which were based on the operators’ subjective opinion, were significantly higher in the residents’ than the specialists’ groups. The intraoperative difficulties required an operator conversion in seven (12.96%) LAs in the residents’ group and 18 (7.32%) LAs in the specialists’ group. All intraoperative difficulties are shown in Table 3; all intraoperative difficulties requiring an operator conversion are shown in Table 4.

**Table 3:** Intraoperative difficulties

Parameter	Group 1(residents)	Group 2 (specialists)	*p* -value
Total	13 (24.07%)	27 (10.98%)	0.0104
Difficulty to achieve good and sufficient working space	4 (7.41%)	4 (1.63%)	0.0547
Intra-abdominal adhesions obstructing performance of the surgery	2 (3.70%)	6 (2.44%)	0.9554
Difficulty in preparation	4 (7.41%)	12 (4.88%)	0.6784
Difficulty in recognizing the anatomy, the layers	2 (3.70%)	2 (0.81%)	0.3068
Difficulty to achieve sufficient hemostasis	1 (1.85%)	3 (1.22%)	0.7732

**Table 4:** Intraoperative difficulties requiring conversion of an operator

Parameter	Group 1(residents)	Group 2 (specialists)	*p* –value
Total	7 (12.96%)	18 (7.32%)	0.1741
Difficulty to achieve good and sufficient working space	2 (3.70%)	3 (1.22%)	
Intra-abdominal adhesions obstructing performance of the surgery	0 (0.00%)	3 (1.22%)	
Difficulty in preparation	2 (3.70%)	7 (2.85%)	
Difficulty in recognizing the anatomy, the layers	2 (3.70%)	2 (0.81%)	
Difficulty to achieve sufficient hemostasis	1 (1.85%)	3 (1.22%)	

We identified 25 cases (8.33%) of operator conversion, a situation in which the operator could not safely complete LA. Operator conversion was a result of an injury of a major vessel or other organ and excessive blood loss in four cases (one case in Group 1 and three cases in Group 2). We found a description of “difficult or insufficient working space” or “massive intra-abdominal adhesions” in the operation reports of eight cases as the possible cause of operator conversion (two in Group 1 and six in Group 2). In the remaining 13 cases, lack of progress in the procedure was due to difficulty in recognizing the anatomical structures or layers or dissection in those layers (four in Group 1, nine in Group 2).

There were two conversions from laparoscopic to classical “open” surgery in Group 2. The first conversion was due to intraoperative difficulties with recognition of anatomical structures and intense bleeding that was impossible to manage laparoscopically. The second was due to an intense bleeding from inferior vena cava that was repaired after conversion with a vein patch from the saphenous vein.

Perioperative complications were observed in 15 (5.0%) patients. Seven patients had perioperative complications of Clavien–Dindo class I, six had class II and two had class III. Detailed characteristics of perioperative morbidity regarding the Clavien–Dindo scale are presented in Table 5. The most common complication in both groups was surgical site infection [two (3.70%) vs. three (1.22%), respectively] and bleeding requiring transfusion [three (1.22%)] in Group 2.

**Table 5:** Perioperative complications

Clavien-Dindo Classification	Parameter	Group 1 (residents)	Group 2 (specialists)	*p*-value
	Total	3 (5.56%)	12 (4.88%)	0.6442
1	Fever of unknown origin	0 (0.00%)	2 (0.81%)	–
1	Surgical site infection	2 (3.70%)	3 (1.22%)	0.4812
2	Bleeding requiring transfusion	1 (1.85%)	3 (1.22%)	0.7732
2	Postoperative pneumonia	0 (0.00%)	2 (0.81%)	–
3	Other organ injury requiring reoperation	0 (0.00%)	1 (0.41%)	–
3	Bleeding requiring reoperation	0 (0.00%)	1 (0.41%)	–

Reoperations were necessary for two patients from Group 2. Both reoperations were performed because of bleeding. In one case, just laparoscopic hematoma removal and control of bleeding from small vessels was performed. In the second case, splenectomy was required.

Five patients (all from Group 2) were readmitted because of complications after discharge. Two of these were readmitted due to pneumonia, two due to a fever of unknown origin, and one due to bleeding from iatrogenic injury of the spleen. The average LOS after surgery for both groups was 2 days. No patients died during the 30-day perioperative period.

All end-points of study for groups 1 and 2 are presented in Table 6.

**Table 6:** End points of the study

Parameter	All	Group 1 (residents)	Group 2 (specialists)	*p* value
Mean operative time (min, ± SD))	103.5 (30–350, SD ±43.5)	106.5 (45–220, SD ± 33.35)	102.8 (30–350, SD ± 45.40	0.5761
Median blood loss (ml, IQR)	50 (20–100) ml	50 (30–100) ml	50 (20–100) ml	0.4325
Intraoperative adverse events, n (%)	26 (8.67%)	5 (9.26%)	21 (8.54%)	0.8643
Complications, n (%)	15 (5.00%)	3 (5.56%)	12 (4.88%)	0.6442
Intraoperative difficulties, n (%)	40 (13.33%)	13 (24.07%)	27 (10.98%)	0.0104
Conversion of the operator, n (%)	25 (8.33%)	7 (12.96%)	18 (7.32%)	0.1741
Conversion to open surgery, n (%)	2 (0.67%)	0 (0.00%)	2 (0.81%)	–
Reoperation, n (%)	2 (0.67%)	0 (0.00%)	2 (0.81%)	–
Readmission, n (%)	5 (1.67%)	0 (0.00%)	5 (2.03%)	–
Mean length of hospital stay (days, ± SD)	1.93 (1–2, SD ±1.11)	1.69 (1–2, SD ±0.97)	1.98 (1–2, SD ±1.14)	0.0422
30-day mortality rate, n (%)	0 (0.00%)	0 (0.00%)	0 (0.00%)	–

## Discussion

During the last three decades, laparoscopy has proven its indisputable advantages in many aspects over open surgery [14, 15]. Access to minimally invasive techniques had increased worldwide. In general, LA is considered a safe operation, although quite technically demanding and with potentially life-threatening morbidity [16–20]. Because of the complexity, advanced minimally-invasive techniques such as LA are still frequently thought to be somehow hazardous if performed by residents. However, there is no evidence supporting this opinion.

The problem of the safety of LA performed by residents in training supervised by an experienced surgeon was addressed. In our study, the course of the LA operation itself did not differ greatly when comparing residents and attending surgeons. The blood loss, conversion rate, and rate of adverse events were similar. Seib and Venkat showed that there was no difference in need of transfusion between the two groups [21, 22]. The risk of complication, readmission or reoperation related to the procedure was also no different. Similar findings have been reported by other authors. Goldfarb and Horesh showed that there were no significant differences between numbers of major complications when comparing residents and surgeons [23, 24]. Notably, Seib showed that resident participation was even associated with decreased odds of serious complications [21]. We noticed a lower incidence of serious perioperative complications than other authors [21, 22].

According to our results, the safety of these procedures performed by residents, regarding the surgery itself and its postoperative course, did not differ from operations performed by attending surgeons. This can likely be explained by our training program, which included liberal active proctoring. The key point into safe LA is training. Most authors agree that the LA learning curve stabilizes between the 20th and 40th surgery [16, 17, 25]. The steepness of a learning curve is related to several factors, including proper theoretical knowledge followed by practical training, the complexity of a procedure, previous experience with other procedures, proctorship and mentoring [21, 22]. According to the literature, the typical learning curve in LA is evaluated in the group of surgeons who already have some experience in advanced laparoscopic techniques, but not in the group of beginners [16, 18]. Another aspect related to the learning curve is the volume of procedures performed in a surgical unit, which should be substantial. Low volume is related to the poor quality of the learning curve [19]. Our department is a referral center for general surgery that specializes in minimally invasive techniques and a teaching hospital with experience in endocrine surgery and advanced laparoscopic procedures. We included several structured training step comprising theoretical background, training on simulators, and assistance in laparoscopic procedures. This is followed by an active participation in basic and advanced operations. We believe that the key point for safe and effective training is active tutoring. We understand this as active participation of a more experienced surgeon who proceeds instead of the prior operator in the most difficult steps of the operation. The incidence of intraoperative difficulties, which was based on the operator’s subjective opinion, was higher in the residents’ than the specialists’ group. More than half of these difficulties enforced an active tutoring and a change of the operator. This parameter was never explored in the field of LA. Notably, the difference in operator conversion between the two groups was not statistically significant and was not related to a higher complication rate. This means that operator conversion was an effective tactic to prevent complications. However, it shows that LA can still be challenging even for specialists.

Another interesting finding is the similar operative time for residents and attending surgeons. Longer operative time seems somewhat natural in the course of a learning curve and for the sake of safety, no time pressure should be put on learning residents. A similar operative time for residents and specialists can be natural when related to the tactic of choosing potentially easier cases for beginners and more difficult (that is, longer-lasting surgeries) for more experienced surgeons. In our department, the tactic of choosing potentially easier cases for residents was not observed. The results were similar to those of Horesh but differ from these of Goldfarb, Seib, and Venkat, who showed that residents operate for longer. We think that this phenomenon could be due to “learning from the experience of the more experienced.” This was described by Geubbels et al., who wrote that some surgeons benefit directly from the experience already gained by more experienced colleagues and then “learn to operate faster.” Furthermore, institutional experience grows parallel to individual experience over time [26]. They confirmed surgical experience as the primary explanation for a decrease in surgical time but he emphasized that as decrease in operating time in case of bariatric procedures after every 50 patients is indeed well explained by an increase in experience, the differences among the surgeons are not [26]. Most attending surgeons at the beginning of our study were specialists just for 2–3 years, so were probably close to their learning curve stabilization point then. The mean operation time in our study was shorter than that in other reports [21–24].

Another interesting finding was that patients operated by residents had shorter LOS. Similarly to our findings, Seib [21] noted that residents participation as an operator has a positive effect on LOS. It is not clear why patients operated by specialists had a statistically significant longer hospital stay. It could be explained, again, by the selection bias resulting in more easy cases operated by residents as shown previously.

Recent surveys showed that surgical residents, even in highly developed countries, are concerned about their training in minimally invasive techniques and conclude that most are unprepared for autonomous laparoscopic operations [27, 28]. Modifications of the residency program are required to fulfill both the needs of residents and expectations of the patients to get a laparoscopic approach to most of the abdominal pathologies. Residents should also be encouraged to use any sort of laparoscopic trainer to further improve their laparoscopic skills. However, safe minimally invasive techniques training should generally be limited to high volume centers.

This study has several limitations. Our institution is a referral center for general surgery specialized in minimally invasive techniques and a teaching hospital. It was impossible to include several variabilities such as the influence of previous experience of residents in minimally invasive techniques. Moreover, it was impossible to include individual skills and natural predispositions in surgical performance. Another limitation is the retrospective nature of the study, which was performed without randomization. Quite naturally for those settings, the group of patients was quite heterogenous and selection bias could impact the results. Therefore, a prospective study should be designed to confirm these findings.

## Conclusions

The results of our study have shown that laparoscopic transperitoneal adrenalectomy performed by a supervised resident is a safe procedure. There is no difference in operating time between residents and specialists as operating surgeons. Patients operated on by residents have shorter LOS in hospital. Liberal use of “operator conversions” from resident to attending surgeon and from a surgeon to a senior surgeon provides reasonable safety and prevents complications.

In the setting of high volume centers of minimally invasive techniques, closed supervision allows residents to safely perform LA.

## Data Availability

The datasets used and/or analysed during the current study are available from the corresponding author on reasonable request.

## Abbreviations

BMI: body mass index
CI: confidence interval
CT: computed tomography
LA: laparoscopic adrenalectomy
LOS: length of hospital stay
MRI: magnetic resonance imaging
OR: odds ratio
PET: positron emission computed tomography
SD: standard deviation

## References

[1] Gagner M, Lacroix A, Bolte E (1992). Laparoscopic adrenalectomy in Cushing’s syndrome and pheochromocytoma. N Engl J Med.

[2] Maccabee DL, Jones A, Domreis J (2003). Transition from open to laparoscopic adrenalectomy: the need for advanced training. Surg Endosc.

[3] Natkaniec M, Pedziwiatr M, Wierdak M (2015). Laparoscopic adrenalectomy for pheochromocytoma is more difficult compared to other adrenal tumors. Wideochir Inne Tech Maloinwazyjne.

[4] Pędziwiatr M, Wierdak M, Ostachowski M (2015). Single center outcomes of laparoscopic transperitoneal lateral adrenalectomy - Lessons learned after 500 cases: A retrospective cohort study. Int Surg.

[5] Zhang X, Wang B, Ma X (2009). Laparoscopic adrenalectomy for beginners without open counterpart experience: initial results under staged training. Urology..

[6] Sefr R, Penka I, Olivero R (1995). The impact of laparoendoscopic surgery on the training of surgical residents. Int Surg.

[7] Itani KM, DePalma RG, Schifftner T, Sanders KM, Chang BK (2005). Surgical resident supervision in the operating room and outcomes of care in veterans affairs hospitals. Am J Surg.

[8] Khuri SF, Henderson WG, Daley J, Jonasson O, Jones RS (2008). Successful implementation of the Department of Veterans Affairs’ National Surgical Quality Improvement Program in the private sector: the patient safety in surgery study. Ann Surg.

[9] Fischer CP, Hong JC (2006). Early perioperative outcomes and pancreaticoduodenectomy in a general surgery residency training program. J Gastrointest Surg: official J Soc Surg Aliment Tract.

[10] Zacharias Mario, Haese Alexander, Jurczok Andreas, Stolzenburg Jens-Uwe, Fornara Paolo (2006). Transperitoneal Laparoscopic Adrenalectomy: Outline of the Preoperative Management, Surgical Approach, and Outcome. European Urology.

[11] Major P, Matlok M, Pedziwiatr M (2012). Do we really need routine drainage after laparoscopic adrenalectomy and splenectomy?. Wideochir Inne Tech Maloinwazyjne.

[12] Dindo D, Demartines N, Clavien PA (2004). Classification of surgical complications: a new proposal with evaluation in a cohort of 6336 patients and results of a survey. Ann Surg.

[13] World Medical Association (2013). World medical association declaration of Helsinki: ethical principles for medical research involving human subjects. JAMA.

[14] Agha R, Muir G (2003). Does laparoscopic surgery spell the end of the open surgeon?. J Royal Society of Med.

[15] Pędziwiatr M, Natkaniec M, Kisialeuski M (2014). Adrenal incidentalomas: should we operate on small tumors in the era of laparoscopy?. Int J Endocrinol.

[16] Fiszer P, Toutounchi S, Pogorzelski R, Krajewska E, Ciesla W, Skórski M (2012). Laparoscopic adrenalectomy – assessing the learning curve. Pol Przegl Chir.

[17] Guerrieri M, Campagnacci R, De Sanctis A, Baldarelli M, Coletta M, Perretta S (2008). The learning curve in laparoscopic adrenalectomy. J Endocrinol Investig.

[18] Goitein D, Mintz Y, Gross D, Reissman P (2004). Laparoscopic adrenalectomy: ascending the learning curve. Surg Endosc.

[19] Treter S, Perrier N, Sosa JA, Roman S (2013). Telementoring: a multi-institutional experience with the introduction of a novel surgical approach for adrenalectomy. Ann Surg Oncol.

[20] Pędziwiatr M, Matłok M, Kulawik J (2013). Laparoscopic adrenalectomy by the lateral transperitoneal approach in patients with a history of previous abdominal surgery. Wideochir Inne Tech Maloinwazyjne.

[21] Seib CD, Greenblatt DY, Campbell MJ (2014). Adrenalectomy outcomes are superior with the participation of residents and fellows. J Am Coll Surg.

[22] Venkat R, Valdivia PL, Guerrero MA (2014). Resident participation and postoperative outcomes in adrenal surgery. J Surg Res.

[23] Horesh Nir, Jacoby Harel, Dreznik Yael, Nadler Roy, Amiel Imri, Dotan Zohar A., Gutman Mordechai, Shabtai Moshe, Rosin Danny (2016). Teaching Laparoscopic Adrenalectomy to Surgical Residents. Journal of Laparoendoscopic & Advanced Surgical Techniques.

[24] Goldfarb M, Gondek S, Hodin R, Parangi S (2010). Resident/fellow assistance in the operating room for endocrine surgery in the era of fellowships. Surgery.

[25] Stefanidis D, Goldfarb M, Kercher KW, Hope WW, Richardson W, Fanelli RD (2013). SAGES guidelines for minimally invasive treatment of adrenal pathology. Surg Endosc.

[26] Geubbels N, de Brauw LM, Acherman YI, van de Laar AW, Wouters MW, Bruin SC (2015). The preceding surgeon factor in bariatric surgery:a positive influence on the learning curve of subsequent surgeons. Obes Surg.

[27] De Win G, Everaerts W, De Ridder D (2015). Laparoscopy training in Belgium: results from a nationwide survey, in urology, gynecology, and general surgery residents. Adv Med Educ Pract.

[28] Qureshi A, Vergis A, Jimenez C (2011). MIS training in Canada: a national survey of general surgery residents. Surg Endosc.

